# Major pathologic response to alectinib in *ALK*-rearranged adenocarcinoma of the lung

**DOI:** 10.1186/s40792-018-0430-7

**Published:** 2018-03-09

**Authors:** Naoko Imanishi, Kazue Yoneda, Akihiro Taira, Yoshinobu Ichiki, Naoko Sato, Masanori Hisaoka, Fumihiro Tanaka

**Affiliations:** 1 Second Department of Surgery (Chest Surgery), University of Occupational and Environmental Health Japan, Kitakyusyu, Japan; 2 Department of Pathology, School of Medicine, University of Occupational and Environmental Health Japan, Kitakyusyu, Japan; 3 Department of Pathology and Oncology, School of Medicine, University of Occupational and Environmental Health Japan, Kitakyusyu, Japan

**Keywords:** ALK, Alectinib, Pathologic response, Adenocarcinoma, Lung

## Abstract

**Background:**

Alectinib is a highly selective tyrosine kinase inhibitor of anaplastic lymphoma kinase (ALK) and provided a significantly prolonged progression-free survival compared with chemotherapy in patients with advanced non-small cell lung cancer (NSCLC) harboring rearrangements of the *ALK* gene. Here, we present the first surgical case of *ALK*-rearranged lung adenocarcinoma with major pathological response in resected specimens after treatment with alectinib.

**Case presentation:**

A 65-year-old female with clinical stage IIIA-N2 ALK-rearranged adenocarcinoma originating from the left lower lobe presented. Involvement of lower para-tracheal node was pathologically confirmed by endobronchial ultrasound-guided biopsy. Alectinib was prescribed, as the patient may not tolerate radiotherapy due to a mental illness. After 3 months’ treatment with alectinib, a remarkable radiological and metabolic response was achieved. The patient did not tolerate further continuation of alectinib treatment, and surgery was performed without any morbidity. Only < 10% tumor cells were viable in all resected specimens, indicating major pathological response to alectinib.

**Conclusions:**

Salvage surgery after alectinib treatment may be safe and effective for initially unresectable NSCLC harboring *ALK*-rearrangements.

## Background

Non-small cell lung cancer (NSCLC) with rearrangement of the *anaplastic lymphoma kinase* (*ALK*) gene is a distinct subtype of lung cancer. Crizotinib, the first approved tyrosine kinase inhibitor (TKI) of ALK, provided a significantly prolonged progression-free survival compared with chemotherapy in patients with advanced *ALK*-rearranged NSCLC [1]. Alectinib is a highly selective ALK-TKI and has showed superior efficacy in two randomized phase 3 studies comparing crizotinib with alectinib [2, 3]. Here, we report the first case of *ALK*-rearranged lung adenocarcinoma with major pathological response in resected specimens after treatment with alectinib, which may provide a pathological rationale for its greater clinical efficacy for advanced patients and may warrant clinical trials of adjuvant treatment of alectinib for resectable *ALK*-rearranged NSCLC patients. In addition, salvage surgery after alectinib treatment for initially unresectable *ALK*-rearranged NSCLC patients may be feasible. This study was approved by the Ethics Committee of the University of Occupational and Environmental Health, Japan, and the written informed consent was taken from the patient.

## Case presentation

**Fig. 1:**
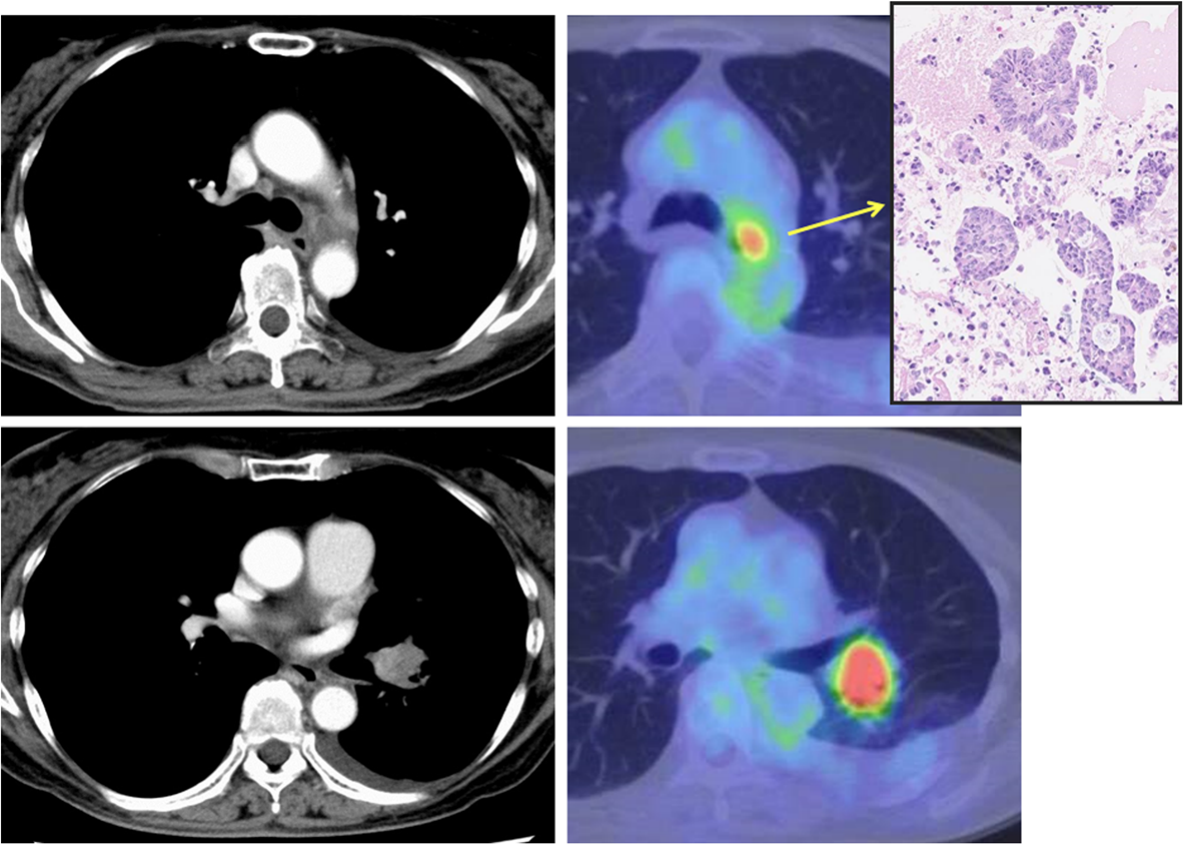
Computed tomography and positron emission tomography before the treatment

A 65-year-old female with a mental illness (bipolar disorder) was referred to our institute for the treatment of *ALK*-rearranged lung adenocarcinoma originating from the left lower lobe. Chest computed tomography (CT) and positron emission tomography (PET) scan revealed enlargement of lower para-tracheal node with a moderate uptake of fluoro-deoxy-glucose (FDG) (Fig. 1), and endobronchial ultrasound-guided biopsy provided pathological evidence of nodal involvement. Whole-body CT/PET scan and brain magnetic resonance imaging revealed no distant metastasis, and her lung adenocarcinoma was diagnosed with clinical stage IIIA-N2 disease.

**Fig. 2:**
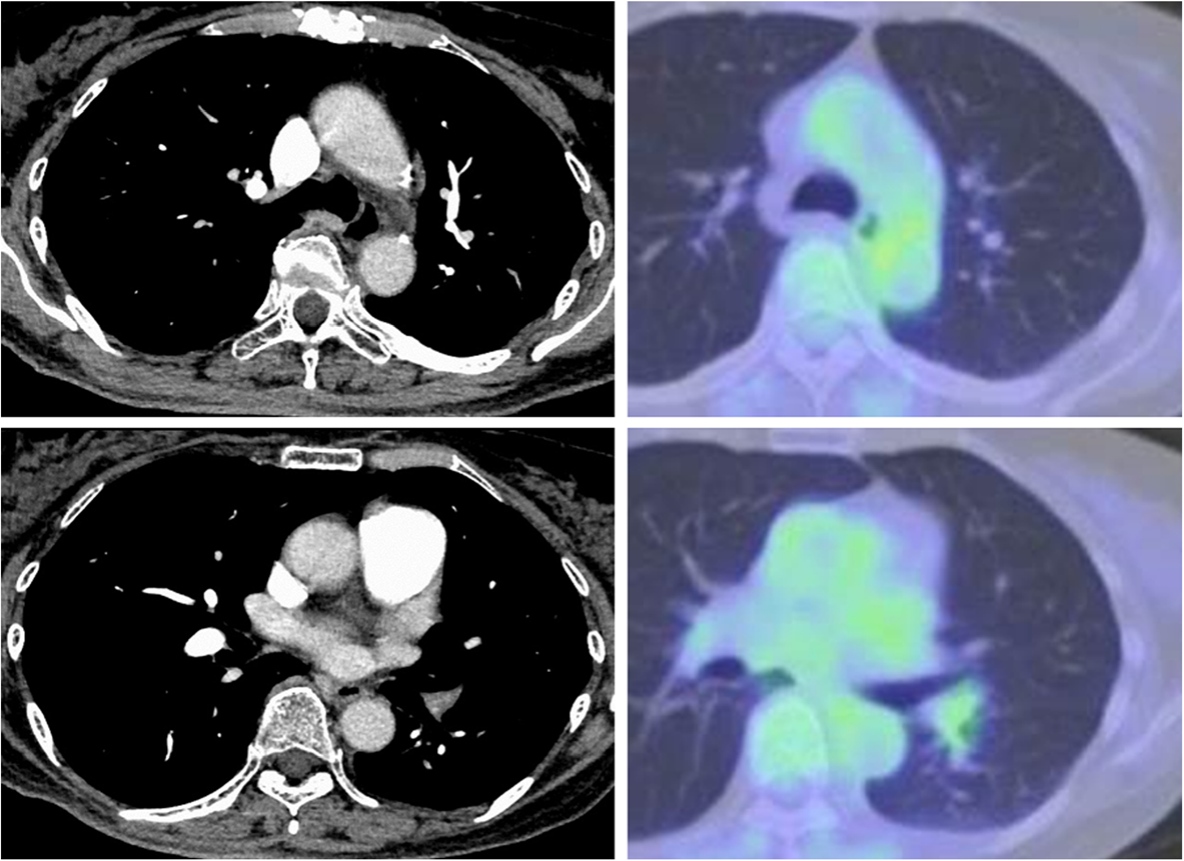
Computed tomography and positron emission tomography after the treatment with alectinib

**Fig. 3:**
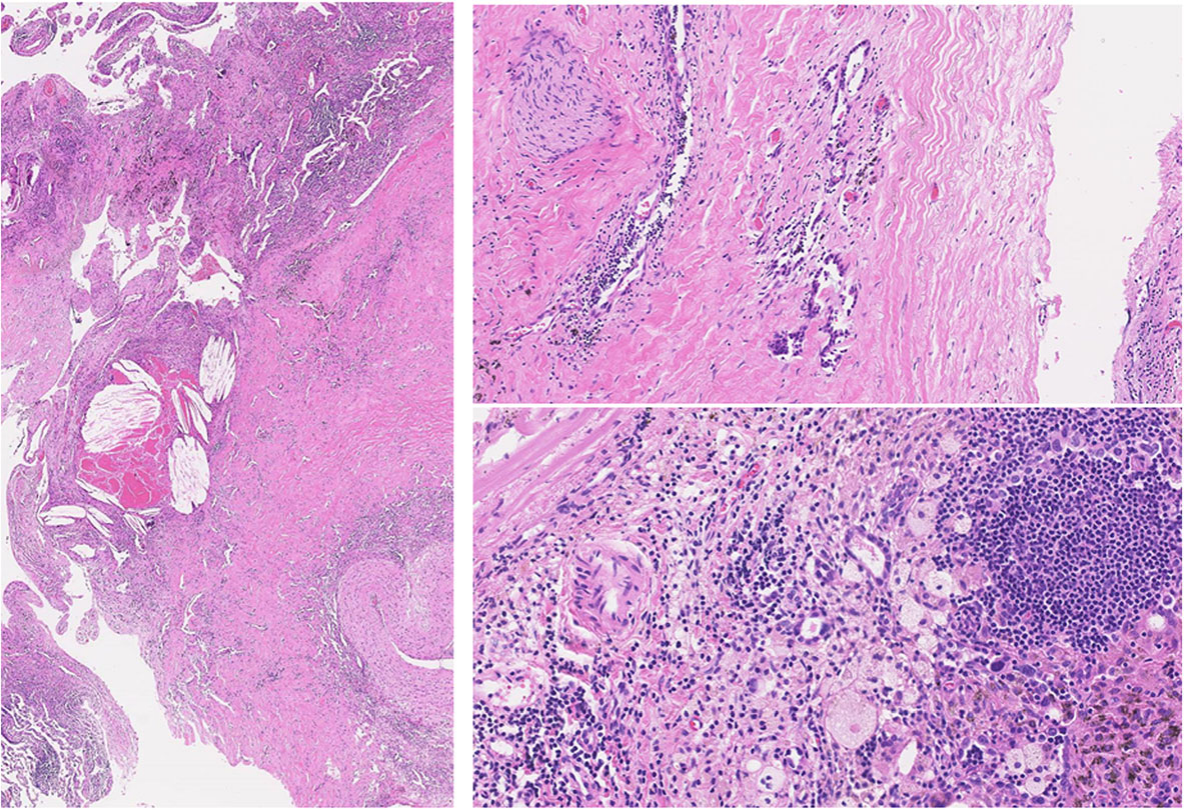
Histological findings after the treatment with alectinib. (Left) No viable tumor cells are seen in most pathological sections cut from the primary tumor and lymph nodes. (Right) Only a small number of atypical cells were seen in some primary tumor sections (upper) and nodal sections (lower)

Alectinib treatment was performed to the patient, as she did not tolerate radiotherapy due to the mental illness. After 3 months’ treatment with alectinib, a remarkable radiological and metabolic response was achieved (Fig. 2). The patient did not tolerate further continuation of alectinib treatment, and surgery was performed. We had continued alectinib treatment until the day before surgery. Complete resection was achieved with combined resection of left lower lobe and lingular segment with systematic nodal dissection, and postoperative course was uneventful. Pathological examination revealed that only < 10% tumor cells were viable in all resected specimens, indicating major pathological response to alectinib (Fig. 3). The patient refused any additional treatment and is alive without any tumor recurrence at 5 months after surgery.

### Discussion

ALK-TKIs usually provide good radiological and metabolic response in most *ALK*-rearranged NSCLC. However, pathological response to ALK-TKIs remains unknown because ALK-TKIs are indicated only for unresectable patients. In the English literature, only two cases showing pathologic response to an ALK-TKI have been reported [4, 5]. In both cases, good radiological and metabolic response was achieved with crizotinib treatment, but pathological specimens revealed only minimal response [4, 5]. In contrast, major pathologic response was achieved in the current case treated with alectinib. These results as well as superior clinical efficacy of alectinib over crisotinib in phase 3 studies [2, 3] may indicate a more potent anti-tumor activity of alectinib.

The major pathologic response achieved with alectinib may warrant clinical trials of alectinib in postoperative adjuvant treatment for resectable *ALK*-rearranged NSCLC patients. A variety of targeting agents such as an antibody against the vascular endothelial growth factor (VEGF) and TKIs of the epidermal growth factor receptor (EGFR) are now tested in clinical trials for resectable NSCLC patients, but these agents can cause unexpected bleeding or delayed wound healing which may lead to increased morbidity and mortality. In contrast, as observed in the present case, ALK-TKIs may not cause such fatal operative complications because ALK targeted by these agents may not express in normal tissues in the adult.

The standard care of treatment for clinical IIIA-N2 NSCLC is chemoradiation, but alectinib treatment was performed in the current case because the patient did not tolerate radiotherapy due to bipolar disorder. After 3 months’ treatment of alectinib, the patient did not even tolerate further continuation of alectinib treatment, and salvage surgery was conducted. We planned to preform only the best supportive care, even when complete resection was not achieved or postoperative recurrence occurred. Fortunately, the postoperative course was uneventful, and the patient is alive without tumor recurrence at 5 months after surgery. The current case may indicate that salvage surgery after alectinib treatment for initially unresectable NSCLC patients is feasible.

## Conclusions

We presented the first case of *ALK*-rearranged lung adenocarcinoma with major pathological response in resected specimens after treatment with alectinib. Salvage surgery after alectinib treatment may be safe and effective for NSCLC harboring *ALK*-rearrangements.

## Abbreviations

ALK: Anaplastic lymphoma kinase
CT: Computed tomography
EGFR: Epidermal growth factor receptor
FDG: Fluoro-deoxy-glucose
NSCLC: Non-small cell lung cancer
PET: Positron emission tomography
TKI: Tyrosine kinase inhibitor
VEGF: Vascular endothelial growth factor
